# Heart disease among Greenlandic children and young adults: a nationwide cohort study

**DOI:** 10.1093/ije/dyac024

**Published:** 2022-02-24

**Authors:** Marie Tindborg, Anders Koch, Mikael Andersson, Klaus Juul, Uka Wilhjelm Geisler, Bolette Soborg, Sascha Wilk Michelsen

**Affiliations:** Department of Epidemiology Research, Statens Serum Institut, Copenhagen, Denmark; Department of Paediatric and Adolescent Medicine, Rigshospitalet University Hospital, Copenhagen, Denmark; Department of Internal Medicine, Queen Ingrids Hospital, Nuuk, Greenland; Department of Infectious Disease Epidemiology and Prevention, Statens Serum Institut, Copenhagen, Denmark; Department of Infectious Diseases, Rigshospitalet University Hospital, Copenhagen, Denmark; Ilisimatusarfik, University of Greenland, Nuuk, Greenland; Department of Epidemiology Research, Statens Serum Institut, Copenhagen, Denmark; Department of Paediatric and Adolescent Medicine, Paediatric Cardiology, Rigshospitalet University Hospital, Copenhagen, Denmark; Department of Internal Medicine, Queen Ingrids Hospital, Nuuk, Greenland; Department of Epidemiology Research, Statens Serum Institut, Copenhagen, Denmark; Department of Epidemiology Research, Statens Serum Institut, Copenhagen, Denmark; Department of Paediatric and Adolescent Medicine, Rigshospitalet University Hospital, Copenhagen, Denmark

**Keywords:** Greenland, Inuit, heart disease, congenital heart disease

## Abstract

**Background:**

The incidences of heart disease (HD) and congenital heart disease (CHD) among Inuit in Greenland (GL) and Denmark (DK) are unknown. This study aims to estimate incidence rates (IRs) of HD and CHD among the young Inuit populations in Greenland and Denmark compared with rates among young non-Inuit populations in the same countries.

**Methods:**

A register-based nationwide cohort including all individuals living in Greenland and Denmark from birth to age <40 years through 1989–2014 was formed. Ethnicity was considered Inuit/mixed if at least one parent was registered as being born in Greenland. Information on HD and CHD hospitalization was obtained from national inpatient registers using ICD-8 and ICD-10 codes.

**Results:**

HD IR was lower among individuals living in Greenland compared with those living in Denmark, [73.35^GL^ (95% confidence interval (CI) 68.07 to 79.03)] vs [88.07^DK^ (95% CI 87.38 to 88.76)], whereas CHD IRs were almost similar in the two countries [IR 34.44^GL^ (95% CI 30.89 to 38.40) vs IR 34.67^DK^ (95% CI 34.24 to 35.10)]. Being of Inuit/mixed ethnicity was associated with an increased risk of both HD and CHD compared with non-Inuit in Greenland and Denmark [adjusted hazard ratio HD 2.07^GL^ (95% CI 1.25 to 3.42)] and CHD [2.92^GL^ (95% CI 1.34 to 6.38)].

**Conclusion:**

HD IR was lower in individuals living in Greenland compared with individuals living in Denmark, whereas the CHD IRs were almost the same for both countries. However, the risk of HD including CHD was higher among individuals of Inuit/mixed ethnicity compared with non-Inuit in both countries, suggesting a role of ethnicity among children and younger adults.

Key Messages

- The overall incidences of heart disease and that of congenital heart disease among Inuit in Greenland and Denmark are unknown.
- We found that the overall incidence of heart disease was lower in Greenland compared with Denmark while incidence of congenital heart disease was the same in the the two countries. Incidences of both heart disease and congenital heart disease were higher among individuals of Inuit/mixed ethnicity compared with non-Inuit in both countries.
- If the observed incidences of heart disease and congenital heart disease in Greenland represent an underestimation of the true rates in Greenland, this should lead to changes in diagnostic approach and registration in the country.
- The higher incidences of heart disease and congenital heart disease in individuals of Inuit/mixed ethnicity in both countries could indicate that genetic factors may play a role in heart disease susceptibility in Inuit, although cultural/environmental factors cannot be ruled out.

## Background

Heart disease (HD) among children and young adults represents a diverse group of cardiac diseases occurring at different times before and after birth. HD can be classified as congenital heart disease (CHD) or acquired heart disease.

Worldwide, CHD is the most common congenital birth defect and the main cause of death in the first year of life.^1–3^ CHD incidences vary around the world and, whereas few specific causes are known,^4^^,^^5^ genetic variations and environmental risk factors seem to play important roles.^3–8^

Although much attention has been paid towards ischaemic cardiac disease among Inuit and the presumptive association with fish oil intake, little is known about CHD incidence among Inuit. However, there are indications that Inuit have higher CHD risk. In 1969, Harvald *et al.* reported higher CHD incidence among Greenlandic Inuit compared with other ethnic groups.^9^ A Canadian register-based study found a four times higher CHD incidence among Inuit children in two regions of Canada compared with the general population of the region of Alberta.^10^

Several factors could contribute to CHD incidence among the Inuit, such as vitamin deficiencies during pregnancy or a specific genetic predisposition altering nutrient status or heart formation.^4^^,^^7^^,^^8^^,^^10–12^ Inadequate consumption of vitamin A and folic acid (essential for a normal embryonic development^10^), attributed to transition from Arctic traditional food to a Westernized diet, has been documented among Canadian and Greenlandic Inuit.^13–16^ Until now, few studies have described and evaluated specific genetic or environmental risk factors among Inuit with CHD.^4^^,^^7^^,^^8^^,^^10^

Studies have indicated both higher and lower incidence of ischaemic heart disease and higher incidence of rheumatic heart disease in indigenous populations compared with non-indigenous populations.^4^^,^^10^^,^^16–22^ There have been no studies reporting on the incidence of overall heart disease (including both CHD and acquired heart disease) in Greenland (GL) among children and young adults; thus overall HD and CHD incidence rates (IRs) in this population in recent years are unknown.

Using nationwide data sources over a 25-year period, the aim of this study was to calculate the IRs of HD in general and for CHD specifically among Inuit children and young adults living in Greenland or Denmark (DK), compared with the non-Inuit populations of the same age living in Greenland and Denmark.

## Methods

### Setting

Greenland is an autonomous part of the Kingdom of Denmark with approximately 56 000 individuals. Most are Inuit (90%)^23^ and 30% of the population live in the capital Nuuk.^24^ The population of Denmark is around 100 times larger; 90% are ethnic Danes and 23% live in the capital area of Copenhagen (year 2014).^25^ Approximately 14 000 Greenlandic Inuit live in Denmark (year 2013).^26^ In both countries, all citizens have free access to health care. In Greenland, this consists of a central hospital in Nuuk, medical centres in main towns in every region and nursing stations in settlements. When needed, patients from Greenland are referred to further medical evaluation and treatment in Denmark. Both countries offer prenatal ultrasound (Greenland at week 16, Denmark at weeks 11–13 and 20–22). Participation rates for prenatal ultrasound in Denmark were 92.4% for weeks 11–13 and 94.4% for the weeks 20–22 (2018).^27^ No data were available for Greenland. Infant mortality and perinatal mortality in Greenland are high compared with Denmark.^28–30^ To increase quality of perinatal care and decrease perinatal mortality and morbidity, at-risk pregnancies were centralized in 2002 in Greenland.^29^

### Data sources

Since 1972, all live-born children and new residents in Greenland and Denmark have been assigned a unique personal identifier through the Civil Registration System. The Civil Registration System allows for individual-level follow-up through national registers and provides information e.g on birthplace, sex, date and place of birth, place of residence and time of death. Since 1987, information on all hospital admissions in Greenland has been registered in the Greenlandic Hospital Discharge Register (GHDR), which contains information on diagnoses and dates of admission and discharge. The GHDR used the 8th Revision of the International Classification of Diseases (ICD-8) from 1987 and ICD-10 from 1996. GHDR was validated in 2011 (unpublished report to the Greenland Self Rule), documenting a stable reporting rate, high register completeness, and accuracy of a number of diagnoses from 1987–2009. Tvermosegaard *et al.* validated specific cardiovascular diagnoses in 2018 and found high correctness.^31^ Using the GHDR, we obtained information on hospitalizations in Greenland. To validate correctness of diagnoses, ventricular septal defect was evaluated. Medical records of all patients with a ventricular septal defect after year 2000 were assessed. Of overall 56 cases, medical records were available in 48 cases and in all of these, ventricular septal defect diagnosis was confirmed, although in one case the correct diagnosis was atrioventricular septal defect.

Information on hospitalizations in Denmark was obtained from the Danish National Patient Registry (DNPR) containing complete data on hospitalizations from 1978.^32^ CHD diagnoses in Denmark have been validated and are continuously validated by the Danish Register of Congenital Heart Disease,^33^ both regionally and nationwide in Denmark.^33–35^ Selected acquired heart disease diagnoses have been validated in the DNPR, and validity varies with different disorders. Previous studies have found a positive predictive value of 93% for atrial fibrillation and 65% for acute coronary syndrome.^32^^,^^36^^,^^37^

Only hospital admissions are included in the GHDR, whereas DNPR also contains data on outpatient contacts from 1994, which are included in this study. Supplementary Table S1 (available as Supplementary data at *IJE* online) show cases that would have been missed if only the GHDR had been used to obtain HD diagnoses in the Greenlandic cohort; 36% of Greenlandic HD cases and 50% of CHD cases were registered only in the DNPR, corresponding to the number of individuals referred from Greenland to Denmark for further evaluation and diagnosis.

### Study design

The study was a nationwide register-based retrospective cohort study including all individuals aged 0 to <40 years living in Greenland or Denmark at least once during the period 1989–2014. Participants were considered to be of Inuit/mixed ethnicity if at least one parent was registered as being born in Greenland. Based on place of residence at time of diagnosis (Greenland/Denmark), participants were categoried as: Inuit/mixed living in Greenland, Inuit/mixed living in Denmark, non-Inuit living in Greenland and non-Inuit living in Denmark. Follow-up was from 1 January 1989 or birth, whichever occurred last, until age <40 years, death, emigration or 31 December 2014, whichever occurred first. Participants were excluded from follow-up at time of emigration from Greenland/Denmark and re-included if returning to Greenland/Denmark.

### Definition of HD

HD hospitalizations were identified through the ICD-8 and ICD-10 systems, and diagnoses relevant to HD as reviewed by an experienced paediatric cardiologist (K.J.) were included. CHD hospitalizations representing a subset of HD hospitalizations were identified through CHD specific diagnoses codes (listed in Supplementary Table S2, available as Supplementary data at *IJE* online). Since CHD is innate, all CHD diagnosis were categorized as CHD irrespectively of prior or subsequent HD diagnoses. To reduce the risk of misclassification (e.g. wrongly coded) and to identify clinically relevant cases of HD, HD hospitalizations or outpatient visits were only included if an individual had: (i) more than one HD admission/outpatient visit; (ii) hospitalization due to HD lasting more than 7 days; or (iii) any CHD hospitalization/outpatient visit. Due to small numbers of specific acquired heart disease diagnoses, all HDs were pooled. In Supplementary Table S5 (available as Supplementary data at *IJE* online) the most common acquired heart disease diagnoses were pooled and evaluated.

### Statistical analysis

HD IRs were calculated as the number of events per 100 000 person-years with 95% confidence intervals (CI). The population size of each country in the given time period served as denominator. Associations between potential risk factors and HD were estimated by hazard ratios (HRs) using Cox regression with age as the underlying time scale and with adjustment for ethnicity (Inuit/mixed or non-Inuit), place of residence and the interaction between sex and age, and stratified by residence at birth (Greenland, Denmark or other) and time period. HRs for individuals living in Greenland and in Denmark were estimated in two separate models. The proportional hazard assumption was evaluated using statistical tests combined with visual inspection of the scaled Schoenfeld residuals in R4.0.2, packages Survival and Survminer. When both the test and the figures suggested a deviation from the Cox proportional hazards assumption in any of the countries, the relevant variables were considered to have an age-specific effect with a cut-point supported by the analyses. The assumption was evaluated in a Cox regression model with age as underlying time scale, with adjustment for sex, ethnicity, place of residence and residence at birth, and stratification by time period. Based on this evaluation it was decided to include the interaction between sex and age and to stratify by place of residence (as this variable was only an adjustment variable). The main effect of sex was estimated in the model without these two changes. Cumulative incidence was estimated as IR per 100 000 person years weighted as the distribution of the world population of the same age and sex in 2015.^38^

## Results

Overall, 69 114 individuals aged 0 to <40 years were included in Greenland and 5 289 211 in Denmark in the period 1989–2014. In Greenland, 53% were men and 85% were of Inuit/mixed ethnicity. In Denmark, 51% were men and 0.4% were of Inuit/mixed ethnicity (Table 1). In Greenland, 690 hospitalizations were due to HD, thereof 324 were CHD. In Denmark, 62 558 hospitalizations were due to HD, thereof 24 627 were CHD (Tables 2 and 3). For lost to follow-up and mortality, see Supplementary Tables S3 and S4 (available as Supplementary data at *IJE* online).

**Table 1:** Demographic characteristics of all individuals aged 0 to <40 years living in Greenland or Denmark from 1989 through 2014

	Greenlandic cohort^a^		Danish cohort^b^	
Characteristics	*N*	(%)^c^	*N*	(%)^c^
All	69 114		5 289 211	
Sex				
Girls/women	32 241	(46.6)	2 591 324	(49.0)
Boys/men	36 873	(53.4)	2 697 887	(51.0)
Ethnicity				
Inuit/mixed^d^	59 018	(85.4)	19 713	(0.4)
Non-Inuit^e^	10 096	(14.6)	5 269 498	(99.6)
Place of residence				
Capital (Nuuk/Capital region of Denmark)	16 235	(23.5)	1 710 873	(32.3)
Town (excluding Nuuk/Capital region of Denmark)	52 879	(76.5)	3 578 338	(67.7)
Year of birth				
1949 to 1959	10 946	(15.8)	842 134	(15.9)
1960 to 1969	13 778	(19.9)	899 861	(17.0)
1970 to 1979	8697	(12.6)	897 250	(17.0)
1980 to 1989	11 186	(16.2)	847 421	(16.0)
1990 to 1999	11 389	(16.5)	801 822	(15.2)
2000 to 2009	8993	(13.0)	695 763	(13.2)
2010 to 2014	4125	(6.0)	304 960	(5.8)

a All individuals living in Greenland at least once between 0 and <40 years of age.

b All individuals living in Denmark at least once between 0 and <40 years of age.

c Percent of included individuals in each cohort.

d At least one parent is born in Greenland.

e Neither of the parents are born in Greenland.

**Table 2:** Crude incidence rates (IRs) and adjusted hazard ratios (HRs) of hospitalization for heart disease (HD)^a^ by demographic characteristics among individuals living in Greenland or Denmark aged 0 to <40 years from 1989 through 2014

	Greenland				Denmark			
	Person-years	Events	IR^b^	HR^c^	Person-years	Events	IR^b^	HR^c^
Characteristics	N	*N*	(95% CI)	(95% CI)	*N*	*N*	(95% CI)	(95% CI)
All	940 742	690	73.35		71 034 207	62 558	88.07	
			(68.07 to 79.03)				(87.38 to 88.76)	
Sex overall								
Girls/women overall	449 799	329	73.14	1 (ref)	34 814 383	29 032	83.39	1 (ref)
			(65.65 to 81.49)				(82.44 to 84.36)	
Boys/men overall	490 944	361	73.53	1.02	36 219 824	33 526	92.56	1.12
			(66.32 to 81.52)	(0.88 to 1.19)			(91.58 to 93.56)	(1.10 to 1.13)
Ethnicity								
Inuit/mixed^d^	860 052	653	75.93	2.07	357 136	395	110.60	1.22
			(70.32 to 81.98)	(1.25 to 3.42)			(100.22 to 122.07)	(1.09 to 1.36)
Non-Inuit^e^	80 690	37	45.85	1 (ref)	70 677 071	62 163	87.95	1 (ref)
			(33.22 to 63.29)				(87.26 to 88.65)	
Place of residence at time of diagnosis								
Capital (Nuuk/Capital region of Denmark)	238 961	146	61.10	1.21	21 944 367	19 708	89.81	1.04
			(51.95 to 71.86)	(1.00 to 1.45)			(88.56 to 91.07)	(1.03 to 1.06)
Town (excluding Nuuk/Capital region of Denmark)	701 782	544	77.52	1 (ref)	49 089 840	42 850	87.29	1 (ref)
			(71.27 to 84.31)				(86.47 to 88.12)	
Age at time of diagnosis								
0 to 2 months	6214	120	1 931.1		412 814	9169	2221.1	
			(1 614.7 to 2 309.4)				(2176.1 to 2 267.0)	
3 to 6 months	6247	29	464.23		417 388	1607	385.01	
			(322.60 to 668.03)				(366.64 to 404.31)	
7 to 11 months	12 342	36	291.69		828 936	1547	186.62	
			(210.41 to 404.38)				(177.55 to 196.16)	
1 to 4 years	99 459	75	75.41		6 671 205	5463	81.89	
			(60.14 to 94.56)				(79.75 to 84.09)	
5 to 9 years	122 887	42	34.18		8 199 675	3019	36.82	
			(25.26 to 46.25)				(35.53 to 38.16)	
10 to 14 years	118 226	47	39.75		8 178 609	2986	36.51	
			(29.87 to 52.91)				(35.22 to 37.84)	
15 to 24 years	211 382	80	37.85		17 256 885	8859	51.34	
			(30.40 to 47.12)				(50.28 to 52.42)	
25 to <40 years	363 987	261	71.71		29 068 697	29 908	102.89	
			(63.51 to 80.95)				(101.73 to 104.06)	

a Heart disease (HD) is defined as any registered heart disease ICD code, all available inpatient and out-patient diagnoses are included. For specific ICD-codes, see Supplementary Table S2 (available as Supplementary data at *IJE* online).

b Incidence rate (IR) relates to the crude incidence of HD per 100 000 person-years.

c Hazard ratio (HR) relates to the risk of having HD.

d At least one parent is born in Greenland.

e Neither of the parents are born in Greenland.

**Table 3:** Crude incidence rates (IRs) and adjusted hazard ratios (HRs) of hospitalization for congenital heart disease (CHD)^a^ by demographic characteristics among individuals living in Greenland and Denmark aged 0 to <40 years from 1989 through 2014

	Greenland				Denmark			
	Person-years	Events	IR^b^	HR^c^	Person-years	Events	IR^b^	HR^c^
Characteristics	*N*	*N*	(95% CI)	(95% CI)	*N*	*N*	(95% CI)	(95% CI)
All	940 742	324	34.44		71 034 207	24 627	34.67	
			(30.89 to 38.40)				(34.24 to 35.10)	
Sex overall								
Girls/women overall	449 799	181	40.24	1 (ref)	34 814 383	12 876	36.98	1 (ref)
			(34.78 to 46.55)				(36.35 to 37.63)	
Boys/men overall	490 944	143	29.13	0.76	36 219 824	11 751	32.44	0.87
			(24.72 to 34.32)	(0.61 to 0.94)			(31.86 to 33.04)	(0.85 to 0.90)
Ethnicity								
Inuit/mixed^d^	860 052	316	36.74	2.92	357 136	174	48.72	1.28
			(32.91 to 41.02)	(1.34 to 6.38)			(41.99 to 56.53)	(1.10 to 1.50)
Non to Inuit^e^	80 690	8	9.91	1 (ref)	70 677 071	24 453	34.60	1 (ref)
			(4.96 to 19.82)				(34.17 to 35.03)	
Place of residence at time of diagnosis								
Capital (Nuuk/Capital region of Denmark)	238 961	66	27.62	1.13	21 944 367	7138	32.53	1.16
			(21.70 to 35.16)	(0.86 to 1.48)			(31.78 to 33.29)	(1.12 to 1.19)
Town (excluding Nuuk/Capital region of Denmark)	701 782	258	36.76	1 (ref)	49 089 840	17 489	35.63	1 (ref)
			(32.54 to 41.53)				(35.10 to 36.16)	
Age at time of diagnosis								
0 to 2 months	6214	113	1 818.4		412 814	8 731	2 115.0	
			(1 512.2 to 2 186.6)				(2 071.1 to 2 159.8)	
3 to 6 months	6247	27	432.21		417 388	1 504	360.34	
			(296.40 to 630.25)				(342.58 to 379.02)	
7 to 11 months	12 342	32	259.28		828 936	1 413	170.46	
			(183.36 to 366.65)				(161.80 to 179.58)	
1 to 4 years	99 459	64	64.35		6 671 205	4 896	73.39	
			(50.37 to 82.21)				(71.36 to 75.47)	
5 to 9 years	122 887	31	25.23		8 199 675	2 057	25.09	
			(17.74 to 35.87)				(24.03 to 26.19)	
10 to 14 years	118 226	14	11.84		8 178 609	1 226	14.99	
			(7.01 to 19.99)				(14.17 to 15.85)	
15 to 24 years	211 382	18	8.52		17 256 885	1 612	9.34	
			(5.37 to 13.52)				(8.90 to 9.81)	
25 to <40 years	363 987	25	6.87		29 068 697	3 188	10.97	
			(4.64 to 10.16)				(10.59 to 11.35)	

a Congenital heart disease (CHD) is defined as the first registered congenital heart disease ICD code, irrespective of other subsequent heart disease diagnosis. For specific ICD-codes, see Supplementary Table S2 (available as Supplementary data at *IJE* online).

b Incidence rate (IR) relates to the crude incidence of CHD per 100 000 person-years.

c Hazard ratio (HR) relates to the risk of having CHD.

d At least one parent is born in Greenland.

e Neither of the parents are born in Greenland.

### Overall IRs

The crude IR of HD hospitalizations was lower in Greenland compared with Denmark [crude IR 73.35^GL^ (95% CI 68.07 to 79.03)] vs [crude IR 88.07^DK^ (95% CI 87.38 to 88.76)] per 100 000 person-years (Table 2). For CHD, the crude IRs were similar in the two countries [crude IR 34.44^GL^ (95% CI 30.89 to 38.40)] vs [crude IR 34.67^DK^ (95% CI 34.24 to 35.10)] per 100 000 person years (Table 3). For acquired heart disease, the crude IR was lower in Greenland compared with Denmark [crude IR 38.91^GL^ (95% CI 35.12 to 43.10)] vs [crude IR 53.40^DK^ (95% CI 52.86 to 53.94)] per 100 000 person years (Supplementary Table S5). In both countries, the highest crude IRs of CHD and HD were observed among the youngest children and were of similar magnitude (Tables 2 and 3).

**Figure 1:**
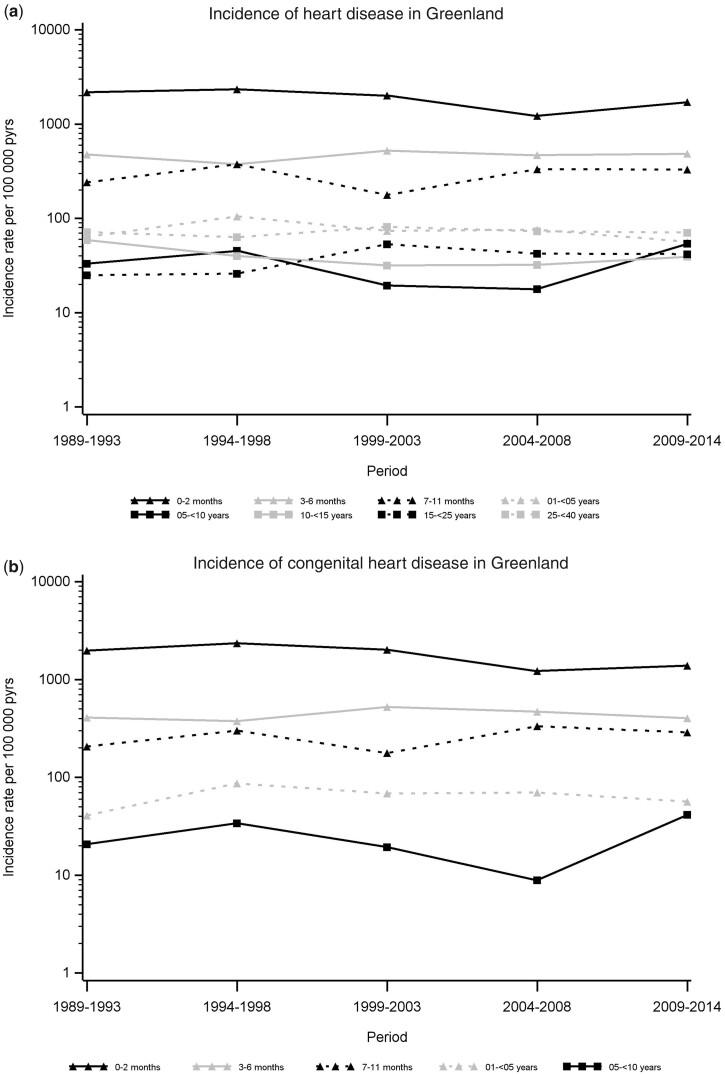
Observed incidence rates (IRs) of hospitalization in Greenland due to heart disease (a) and congenital heart disease (b), during 1989–2014. IRs (per 100 000 person-years) are illustrated as point estimates in 4-year intervals on a logarithmic scale by age groups. For Denmark, see Supplementary Figures S1 and S2 (available as Supplementary data at *IJE* online)

Figure 1 shows the observed IRs per 100 000 person-years of HD and CHD hospitalizations in Greenland. For CHD, only individuals aged <10 years are included in the figure as clinically relevant CHD would have been diagnosed before age 10 years. The highest IRs by age were observed among the youngest children aged <1 year for both HD and CHD, with the highest IRs among children aged 0 to 2 months, the majority of diagnoses being CHD. For both HD and CHD, steady IRs were observed through the study period for all age groups, except for children aged 5 to <10 years, where we observe a slightly increased IR from 2004 through 2014 (Figure 1). For observed HD and CHD hospitalization IRs in Denmark, see Supplementary Figures S1 and S2. 

**Figure 2:**
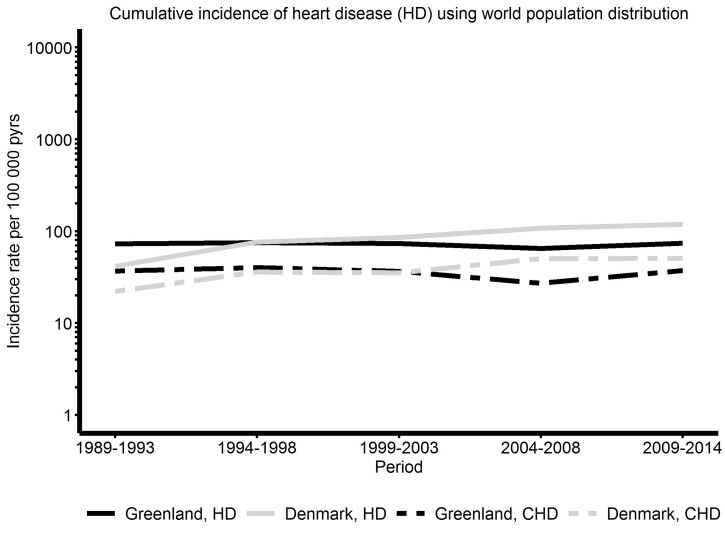
Cumulative incidence rates (IRs) of hospitalization due to heart disease (HD) and congenital heart disease (CHD) among individuals aged 0–<40 years during the period 1989–2014 in Greenland and Denmark. The cumulative IR is weighted as the distribution of the world population of the same age in 2015 and is illustrated as IR per 100 000 person-years on a logarithmic scale

The weighted cumulative incidence of HD was practically steady for Greenland over the entire study period, but increased in Denmark from a level below the IR in Greenland at study start to a level just above the IR in Greenland from 1994–2014 (Figure 2). The same trends were observed for CHD (Figure 2).

### HD incidence by demography

In Greenland, there was no difference in risk of HD hospitalization by sex whereas in Denmark, boys/men had a higher risk of HD than girls/women (Table 2). When taking effect modification by age into account, we found no difference in risk of HD by sex for the youngest individuals (0 to 27 years), but a higher risk of HD for men aged 27 to <40 compared with women of the same age in both countries (Supplementary Table S6, available as Supplementary data at *IJE* online). For CHD, boys/men had a lower risk of hospitalization due to CHD compared with girls/women in both countries (Table 3), but when taking effect modification by age into account, only boys aged 0 to 4 years in Greenland and boys/men aged 4 to <40 years in Denmark had lower risk of CHD compared with girls/women of the same ages, respectively (Supplementary Table S6; also see Supplementary Table S7, available as Supplementary data at *IJE* online, for specific CHD diagnoses by sex).

Living in the capital compared with living outside the capital at time of diagnosis was just associated with a higher risk of HD hospitalization in both Greenland [HR 1.21^GL^ (95% CI 1.00 to 1.45)] and Denmark [HR 1.04^DK^ (95% CI 1.03 to 1.06)], (Table 2). However, there was no difference in risk of CHD hospitalization by place of residence in Greenland (Table 3). See Supplementary Table S8 (available as Supplementary data at *IJE* online) for the specific diagnoses by place of residence.

For both countries, being of Inuit/mixed ethnicity was associated with an increased risk of HD compared with being non-Inuit (Table 2), whereas the risk of CHD by ethnicity was approximately 2-fold higher in Greenland [HR 2.92^GL^ (95% CI 1.34 to 6.38)] than in Denmark [HR 1.28^DK^ (95% CI 1.10 to 1.50)], (Table 3).

### Sensitivity analyses

**Figure 3:**
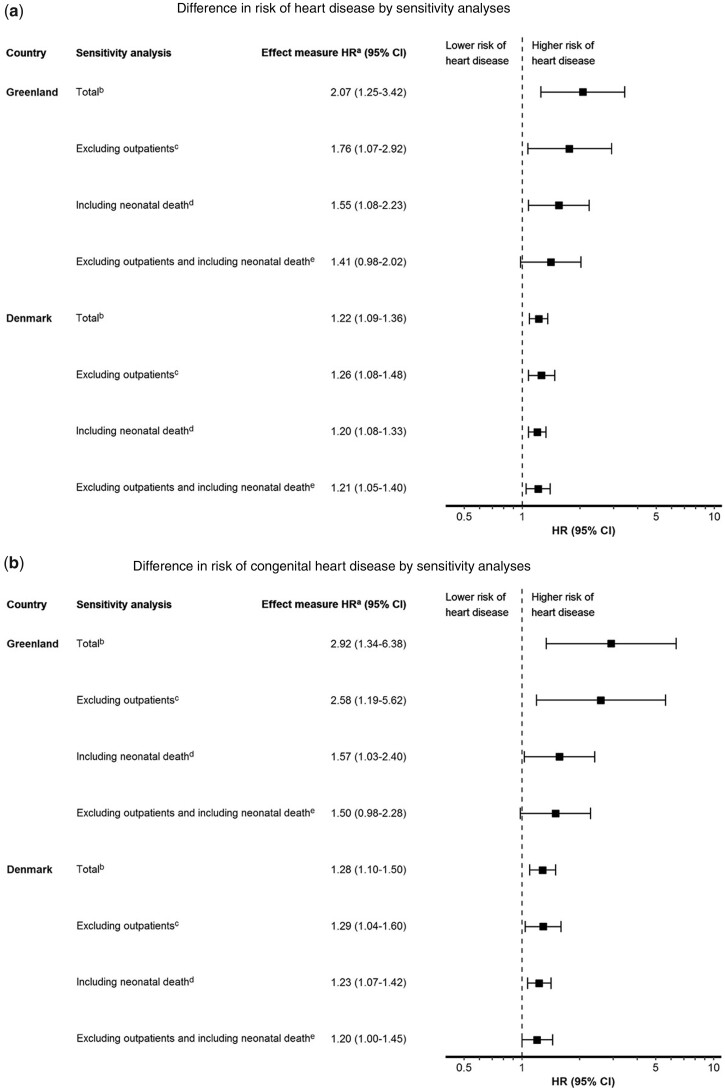
Differences in risk of (a) heart disease (HD) and (b) congenital heart disease (CHD) among individuals of Inuit/mixed ethnicity compared with non-Inuit for Greenland and Denmark, by sensitivity analysis (see Supplementary Tables S9–S14 for all performed analyses). a) Hazard ratio (HR) relates to the risk of having HD/CHD. b) Total is the baseline risk of HD/CHD among individuals of Inuit/mixed ethnicity compared with non-Inuit (see Tables 2 and 3 for all data) prior to sensitivity analysis. c) The risk of HD/CHD among individuals of Inuit/mixed ethnicity compared with non-Inuit, when excluding outpatient diagnosis (see Supplementary Tables S9 and S10, available as Supplementary data at *IJE* online for all data). d) The risk of HD/CHD among individuals of Inuit/mixed ethnicity compared with non-Inuit, when including neonatal death (death before age 31 days) as HD/CHD (see Supplementary Tables S11 and S12, available as Supplementary data at *IJE* online for all data). e) The risk of HD/CHD among individuals of Inuit/mixed ethnicity compared with non-Inuit, excluding outpatient diagnosis and including neonatal death as HD/CHD (see Supplementary Tables S13 and S14, available as Supplementary data at *IJE* online for all data).

Possible effect modification was assessed by: (i) excluding outpatient diagnoses, as 17% of HD diagnoses in Denmark were outpatient diagnoses (Supplementary Tables S9 and S10, available as Supplementary data at *IJE* online); (ii) including neonatal death (before age 31 days) as CHD cases (Supplementary Tables S11 and S12, available as Supplementary data at *IJE* online); and (iii) both (i) and (ii) (Supplementary Tables S13 and S14, available as Supplementary data at *IJE* online). Figures 3a and b show the differences in risk of HD and CHD among individuals of Inuit/mixed ethnicity compared with non-Inuit by sensitivity analyses for both countries (for all sensitivity analyses, see Supplementary Tables S9–S14, available as Supplementary data at *IJE* online). When excluding outpatient diagnoses or including neonatal death as HD/CHD, being of Inuit/mixed ethnicity was still associated with an increased risk of HD/CHD, respectively, when compared with non-Inuit in both countries. When including neonatal death as CHD, CHD HR for individuals of Inuit/mixed ethnicity in Greenland is markedly attenuated, most likely due to high neonatal mortality rates among both individuals of Inuit/mixed ethnicity and non-Inuit (for mortality rates see Supplementary Table S4, available as Supplementary data at *IJE* online). When excluding HD and CHD outpatient diagnoses (Supplementary Tables S9 and S10), HD and CHD IRs in Greenland (and Denmark) were actually higher outside the capital, as was HD HR, leading us to believe that inhabitants outside the capital are diagnosed at least as frequently as inhabitants in the capital living closer to the most specialized hospital. 

## Discussion

To our knowledge, this register study is the first to estimate HD and CHD incidences specifically in children and young adults in Greenland. We found the following results: the HD IR in children and young adults aged 0 to <40 years during 1989–2014 was lower in Greenland compared with Denmark, whereas CHD IRs were the same in the two countries. Both HD and CHD IRs in Greenland remained at the same levels, but the IRs for both increased in Denmark during the study period.

HD and CHD HRs were higher among individuals of Inuit/mixed ethnicity compared with non-Inuit in both countries. Being of Inuit/mixed ethnicity was associated with approximately 2-fold higher risk of CHD in Greenland compared with Denmark. Changes in registration practice, genetic variation and environmental risk factors, or a combination of these, may explain the findings.

### Registration practices and health care

Our findings relied on register-based diagnoses and thus were susceptible to differences in register and referral practices, access to health care and changes in clinical practice.^39^ Worldwide, CHD IR is very stable over time.^5^ Therefore, although lower than in Denmark, the observed stable CHD IR in Greenland is not surprising. The lower observed HD IR in Greenland compared with Denmark may be caused by a general underestimation of HD in Greenland due to, for example, death before diagnosis or less access to hospital and to specialized diagnostic tools. Also, in Greenland only hospital admissions were included, whereas in Denmark also outpatient visits (17%), where less severe HD cases are typically seen, were included. In fact when excluding outpatient diagnoses, IRs for both HD and CHD were higher in Greenland than in Denmark. Furthermore during the study period, easier access to echocardiography and the advances in medical equipment available in Denmark than in Greenland could have led to higher rates of clinically less relevant and less severe HD diagnoses (e.g. small ventricular septal defects) in Denmark. Also, a lowered threshold for referral to hospitals in the period could have led to increasing HD IR in Denmark throughout the study period. Second, only week 16 ultrasound is available in Greenland. Very few structural heart diseases can be recognized at week 16 ultrasound, if performed by an experienced obstetrician with specialty in prenatal screening. We therefore believe that the number of late abortions due to CHD found by prenatal ultrasound in Greenland is low, which may also contribute to higher CHD risk among individuals of Inuit/mixed ethnicity in Greenland. Third, infant mortality before age 1 year is higher in Greenland compared with Denmark; this may lead to underestimation of CHD risk in Greenland due to death before diagnosis.^28^^,^^30^^,^^40^^,^^41^ To evaluate this, we included neonatal death as CHD, which showed overall higher HD and CHD IRs in Greenland compared with Denmark. However, neonatal death may also be due to higher burden of infectious diseases, limited access to highly specialized neonatal care, or genetic factors.^4^^,^^5^^,^^28^^,^^29^^,^^42^ Thus, in spite of our observations of lower HD and CHD IRs among Greenlanders, the true HD and CHD IRs among Greenlandic children may in fact be at the same level or higher than among Danish children. In comparison Arbour *et al.* found that congenital heart defects were increased [odds ratio (OR) of 4.18, 95% CI 3.2 to 5.4] for diagnoses of bulbus cordis anomalies and anomalies of cardiac septal closure, obtained by chart review among Inuit children between 1989 and 1994, compared with the general population of the region of Alberta.^10^ Using chart reviews in contrast to our use of national register-based discharge diagnoses and three diagnostic criteria, Arbour *et al*. may have identified more cases, but addressed only regional coverage in Arctic Canada for a shorter study time period than the present study. The observed differences of an approximately 2-fold higher CHD risk among individuals of Inuit/mixed ethnicity in Greenland compared with Denmark may most likely be ascribed to the factors discussed above.

### Acquired heart disease

Rheumatic heart disease is the most common cause of acquired heart disease in children throughout the developing world.^43^^,^^44^ Recent screening studies among Aboriginal Australians and other high-risk populations have reported a surprisingly high prevalence of rheumatic heart disease of 1.5–5.7% among schoolchildren.^19–21^ Due to low numbers of rheumatic heart disease cases, we were not able to evaluate rheumatic heart disease in Greenland in this study. Due to this and varying validity of acquired heart disease diagnoses in the DNPR,^36^^,^^37^ further evaluation of incidence and risk of specific acquired heart disease diagnoses (e.g. heart valve disease) in Greenland and Denmark among children and young adults might need to be supplemented by prevalence studies using additional echocardiography screening,^45^ to avoid risk of underestimation.

### Genetic variations and sex

HD and CHD risks were higher among individuals of Inuit/mixed ethnicity compared with non-Inuit in both countries, irrespective of sensitivity analysis. This is also seen in Canada, where CHD IR was found to be higher among Inuit compared with non-Inuit by Arbour *et al.*^10^ Although >80% of Greenlanders have some European ancestry, Inuit appear to be a genetically distinct group, possibly more vulnerable to some metabolic diseases such as hyperlipidaemia, diabetes type 2 and obesity than other populations.^4^^,^^16^^,^^46^ However, as our study population is young we find it less likely that genetic variants promoting metabolic lifestyle diseases may explain the observed ethnic differences in HD and CHD risk in our study. Yet, other genetic factors than those acting through metabolism could partly explain this difference.^11^ Studies on genetic CHD disposition in Inuit are warranted, but to our knowledge no such studies have yet been published.^4^ The finding that individuals of Inuit/mixed ethnicity in our study had higher HD risk irrespective of country could indicate that genetic factors may play a role in HD susceptibility in Inuit.

A few studies have described higher incidence of CHD among girls/women compared with boys/men, but data are not consistent on included CHD diagnoses.^47^^,^^48^ Some CHDs are related to sex-specific syndromes, such as coarctation of the aorta and Turner’s syndrome,^49^ but based on clinical experience the number of cases due to such CHD-prone syndromes is expected to be very low in this study (personal communication, Department of Paediatric Cardiology, Rigshospitalet, Copenhagen, Denmark). In contrast, a possible explanation for the sex inequality in CHD IR could be that infant mortality is approximately two times higher among boys in Greenland/Denmark compared with girls ^28^^,^^40^^,^^41^ (see Supplementary Table S4). If boy infants with possible undiagnosed CHD would die at a higher rate than girl infants, this might explain the observed higher IR among girls than boys.

### Changes in environmental risk factors

With stable HD and CHD IRs in Greenland, we find it less likely that changes in environmental risk factors in the country during the study period have been of importance. Our initial assumption was that the majority of individuals of Inuit/mixed ethnicity in Denmark live like Danes. If this were true, we would expect to find a difference in HD risk between individuals of Inuit/mixed ethnicity in Greenland compared with those living in Denmark. In Greenland, living conditions for the majority of non-Inuit (who are mainly Danes) may be of a higher standard than for individuals of Inuit/mixed ethnicity, primarily due to higher income. This may create a healthy worker effect. As the health system in Denmark is markedly more specialized than in Greenland, the same difference between Inuit/mixed and non-Inuit in Denmark could also represent an unhealthy patient effect, generated by an assumption that a majority of individuals of Inuit/mixed ethnicity who migrate to Denmark have poor health compared with the general Danish population (e.g. they migrate due to health issues such as HD).

### Strengths and limitations

Our study has several strengths. The study is population-based over a 25-year period. The health care systems in the two countries are interconnected and clinical practices are similar. Inclusion/censoring and information on demographic characteristics were based on information from national registers with mandatory registration, reducing selection and information bias. There is no international standard definition for CHD,^35^ but all diagnoses were assessed by an experienced paediatric cardiologist. Thereby, clinically unspecific diagnoses were discarded (e.g. heart murmur). Furthermore, ventricular septal defect cases registered in the GHDR were validated through assessment of medical records, which showed that 86% of the diagnoses were correct. In addition, cardiovascular diagnoses in the GHDR were validated in 2018, where >90% of discharge diagnoses were found to be correct,^31^ and CHD diagnoses in DNPR have likewise been validated.^33–35^^,^^50^ Comparison of HD IRs between the countries was done using the cumulative HD IR during the study period, using the World Health Organization age distribution to ensure that variation in population distribution did not affect our results.

Being register-based, our data were susceptible to changes in register practice, referral practices, access to health care and clinical practices (e.g. pulse oximetry screening^39^) etc., which might lead to differences in HD patterns in national registers. This might lead to both under- and overestimation of clinically significant HD. To avoid misclassification due to transient neonate cardiac conditions, we excluded all DP diagnoses (‘certain conditions originating in the perinatal period DP00-DP96’). However, preterm babies with persistent ductus arteriosus or atrial septal defect/persistent foramen ovale may still be included in the study as CHD, but as this potential misclassification would most likely be non-differential and a rare event, the bias on HRs will be negligible. Last, the population size in the Greenlandic cohort was much smaller compared with the Danish cohort, limiting statistical power. However the population of Greenland, with inclusion of all individuals in the relevant age group, determines our sample size in the Greenlandic cohort; and in all analysis, effect of adjustment variables depending on country, is taken into consideration.

In conclusion, we showed that over a 25-year period the HD incidence in children and young adults was lower in Greenland compared with Denmark, whereas the incidence of CHD was the same. In Greenland, HD and CHD IRs remained at the same levels during the study period, but both increased in Denmark. In both countries, individuals of Inuit/mixed ethnicity had higher risk of HD and CHD than non-Inuit.

If the observed HD and CHD incidences in Greenland (vs in Denmark) represent an underestimation of the true rates in Greenland, this should lead to changes in diagnostic approach to and registration of HD in the country. The higher observed risk of HD and CHD in individuals of Inuit/mixed ethnicity in both countries indicate that genetic factors may play a role in HD and CHD risk.

## Ethics approval

The study was purely register-based and did not physically involve any individuals. The study was conducted in accordance with the Helsinki II Declaration and was approved by the Research Ethics Committee for Health Science Research in Greenland (approval No. 2017–2107). The study was reported to the Danish Data Protection Agency and the Greenlandic Health Authorities.

## Data availability

All relevant data are presented in the manuscript and in the Supplementary data. Due to restrictions, exact numbers for less than five cases could not be presented. Data are available from the authors at Statens Serum Institut [swm@ssi.dk], after clearance from the Research Ethics Committee for Health Science Research in Greenland, for researchers who meet the criteria for access to confidential data.

## Supplementary data

Supplementary data are available at *IJE* online

## Author contributions

All authors made substantial contributions to the acquisition, analysis and interpretation of the data, and drafting, revising and approving the final version of the paper, and all agreed to be accountable for all aspects of the work.

## Funding

This work was supported by: Sundhedspuljen Greenland Self-Government (No. 2017–3018), Greenland Research Council (Nanoq–ID nr.: 6597334) and Dagmar Marshalls Fond. The sponsors played no role in the study design, the collection, analysis or interpretation of data, the writing of the report or the decision to submit the manuscript for publication.

## Supplementary Material

dyac024_Supplementary_Data
