# Supplementary material for: Heart disease among Greenlandic children and young adults: a nationwide cohort study
Source: Int J Epidemiol. 2022 Feb 24;51(5):1568–80. doi: 10.1093/ije/dyac024 (PMC9558066; doi:10.1093/ije/dyac024)
Supplement: dyac024_Supplementary_Data [file dyac024_supplementary_data.pdf]

SUPPLEMENTARY DATA: TABLES AND FIGURES

Heart disease among Greenlandic children and young adults: A nationwide cohort study

M. Tindborg, A. Koch, M. Andersson, K. Juul, U. W. Geisler, B. Soborg, and S. W. Michelsen

Supplementary Table S1: Cases of HD and CHD by country of residence at time of diagnosis and by register, corresponding to number of individuals referred from Greenland to Denmark for further evaluation for a heart disease and where heart disease was confirmed

	Greenland<br>N (%)	Denmark<br>N (%)
**Heart disease**	**690 (100)**	**62 568 (100)**
Diagnosed in GHDR[a]	442 (64)	<5 (<.01)
Diagnosed in DNPR[b,c]	248 (36)	62 554 - 62 557 (100)[d]
**Congenital heart disease**	**324 (100)**	**24 627 (100)**
Diagnosed in GHDR[a]	161 (50)	0 (0)
Diagnosed in DNPR[b,d]	163 (50)	24 627 (100)

[a]GHDR= Greenlandic Hospital Discharge Register. [b]DNPR= Danish National Patient Registry.[c]*In Greenland, 36% of HD diagnoses were obtained from the DNPR.* [d]*For CHD specifically, 50% of the diagnosis were obtained from the DNPR, corresponding to the number of individuals referred from Greenland to Denmark for further evaluation for a heart disease and where a heart disease was confirmed.* [d]Due to restrictions the exact number could not be presented

Supplementary Table S2: Categorisation of hospitalisation discharge diagnoses for heart disease and congenital Heart disease by ICD 8 and ICD 10 codes

**Categories**	ICD-8	ICD-10
ICD codes for all heart disease (including ICD codes for congenital heart disease)	07429, 09392, 391-398, 40019, 402, 404, 410-414, 420-429 *(not 42601, 42710, 42795, 42796*), 746-74739, 760- 76019, di0278249	DI01-02 *(not DI02 and DI029)*, DI05-09, DI11, DI13, DI20-25, DI278A, DI30-471C, DI471G-490BA, DI491E-DI492, DI495-DI515B, DI517-517C, DI518A-B, DI520-521B, DI528A, DQ20-240, DQ242-264
Specific ICD codes for congenital heart disease	746-74739, 760- 76019	DI424A, DQ20-240, DQ242-264
Specific ICD codes for selected acquired heart disease		
Overall	All participants not included as CHD, please see methods section	
Any valve disease	DI011, DI05-DI08, DI33-DI39	391, 394-397, 421, 424
Any ischemic heart disease	DI20-DI25	410-414
Any conduction anomaly	DI44-DI45, DI48, DI49, DI47	
Paroxysmal tachycardia	DI47	

Supplementary Table S3: Lost to follow up by number and percentage (%) in Greenland and Denmark during the study period from 1989 through 2014

	**Greenlandic cohort (%)**	**Danish cohort (%)**
**Migration**	2 964 (4.3)	519 799 (9.8)
**Age>40**	26 292 (38.0)	2 020 438 (38.2)
**Death**	2 683 (3.9)	44 811 (0.8)

Supplementary Table S4: Incidence of death in Greenland and Denmark by sex, ethnicity, and age among individuals living in Greenland or Denmark aged 0-<40 years from 1989 through 2014

Characteristics	Greenland Person years[a] N	Events N	IR[b] (95% CI)	Denmark Person years[a] N	Events N	IR[b] (95% CI)
**Sex**						
Boys/men	495	1 755	3.54 (3.38-3.71)	36 584	29 730	0.81 (0.80-0.82)
Girls/women	455	797	1.75 (1.63-1.88)	35 145	15 266	0.43 (0.43-0.44)
**Ethnicity and age**						
**Inuit/Mixed**						
0 to <31 days	2	320	160.68 (144.00-179.28)	1	32	39.52 (27.95-55.88)
31 days to 4 years	117	249	2.14 (1.89-2.42)	49	36	0.73 (0.53-1.02)
5 to 14 years	233	148	0.64 (0.54-0.75)	99	35	0.36 (0.26-0.50)
15 to 39 years	517	1 756	3.39 (3.24-3.56)	213	414	1.94 (1.76-2.14)
**Non-Inuit**						
0 to <31 days	<1	19	161.07 (102.74-252.51)	140	5 927	42.40 (41.33-43.49)
31 days to 4 years[c]	-	-	-	8 234	3 926	0.48 (0.46-0.49)
31 days to 14 years[c]	18	8	0.46 (0.23-0.91)			
5 to 14 years				16 460	2 036	0.12 (0.12-0.13)
15 to >40 years	63	52	0.82 (0.62-1.08)	46 533	32 590	0.70 (0.69-0.71)

[a]Per 1 000 person years. [b]Incidence rate (IR) relates to the crude incidence of death per 1 000 person years. [c]Small numbers required combined age groups for Non-Inuit in Greenland (31 days to 14 years).

Supplementary Table S5: Crude incidence rates (IRs) of hospitalisations for most common acquired heart disease[a] by ethnicity, age, and country among individuals living in Greenland or Denmark aged 0-<40 years from 1989 through 2014[*]

Characteristics	Greenland Person years N	Events N	IR[b] (95% CI)	Denmark Person years N	Events N	IR[b] (95% CI)
**All HR**	940 742	690	73.35 (68.07-79.03)	71 034 207	62 558	88.07 (87.38-88.76)
**All CHD**	940 742	324	34.44 (30.89-38.40)	71 034 207	24 627	34.67 (34.24-35.10)
**<u>All acquired heart disease</u>**	940 742	366	38.91 (35.12-43.10)	71 034 207	37 931	53.40 (52.86-53.94)
Any valve disease[c]	940 742	87	9.25 (7.50-11.41)	71 034 207	3 375	4.75 (4.59-4.91)
Any ischemic heart disease[c]	940 742	55	5.85 (4.49-7.62)	71 034 207	8 775	12.35 (12.10-12.61)
Any conduction anomaly[*] incl. paroxysmal tachycardia[*,c]	740 511	112	15.12 (12.57-18.20)	57 196 684	16 810	29.39 (28.95-29.84)
Paroxysmal tachycardia[*,c,d]	740 511	58	7.83 (6.06-10.13)	57 196 684	9 386	16.41 (16.08-16.75)
**Ethnicity**						
Inuit/mixed[d]	860 052	337	39.18 (35.22-43.60)	357 136	221	61.88 (54.24-70.60)
Non-Inuit[e]	80 690	29	35.94 (24.98-51.72)	70 677 071	37 710	53.36 (52.82-53.90)
**Age at time of diagnosis**						
0-<18 years	427 953	90	21.03 (17.10-25.86)	29 727 105	5 701	19.18 (18.69-19.68)
>18-<40 years	512 789	276	53.82 (47.83-60.56)	41 307 102	32 230	78.03 (77.18-78.88)

[*]Conduction anomalies and paroxysmal anomalies are only evaluated from 1994 and forward, as these diagnoses were not part of ICD8.
[a] Acquired heart disease is defined as any registered acquired heart disease ICD code not included as CHD, please see methods section. For specific ICD-codes, see Supplementary Table S2
[b]Incidence rate (IR) relates to the crude incidences of HD/CHD/acquired heart disease/specific acquired heart diseases per 100 000 person years.
[c]A participant can only contribute to one acquired heart disease IR estimate. Acquired heart diseases are prioritised as follows 1) valve disease, 2) ischemic heart disease, and 3) paroxysmal tachycardia. [d]At least one parent is born in Greenland. [e]None of the parents are born in Greenland.

Supplementary Table S6: Overall and age specific Hazard Ratios (HRs) of hospitalisation for heart disease (HD)[a] and congenital heart disease (CHD)[b] by sex among individuals living in Greenland or Denmark aged 0-<40 years from 1989 through 2014

	Greenland	Denmark
	HR[c] (95% CI)	HR[c] (95% CI)
**Sex overall[c]**		
Girls/women	1 (ref)	1 (ref)
Boys/men	1.02 (0.88-1.19)	1.12 (1.10-1.13)
**Sex by age (days/years)[c]**		
**≤ 9 700 days/26.6 years**		
Girls/women	1 (ref)	1 (ref)
Boys/men	0.90 (0.75-1.09)	1.02 (0.99-1.04)
**> 9 700 days/26.6 years**		
Girls/women	1 (ref)	1 (ref)
Boys/men	1.30 (1.00-1.68)	1.25 (1.22-1.28)
**Congenital heart disease**		
**Sex overall[c]**		
Girls/women overall	1 (ref)	1(ref)
Boys/men overall	0.76 (0.61-0.94)	0.87 (0.85-0.90)
**Sex by age, (days/years)[c]**		
**≤ 1 500 days/4.1 years**		
Girls/women	1 (ref)	1 (ref)
Boys/men	0.74 (0.57-0.97)	1.00 (0.97-1.03)
**> 1 500 days/4.1 years**		
Girls/women	1 (ref)	1 (ref)
Boys/men	0.81 (0.54-1.21)	0.68 (0.65-0.71)

[a]HD is defined as any registered heart disease ICD code, all available in-patient and out-patient diagnoses are included. For specific ICD-codes, see Supplementary Table S2. [b]CHD is defined as the first registered congenital heart disease ICD code, irrespectively of other subsequent heart disease diagnosis, all available in-patient and outpatient diagnoses are included. For specific ICD-codes, see Supplementary Table S2. [c]Hazard ratio (HR) relater to the risk of having HD. The assumption was evaluated in a Cox regression model with age as underlying time scale, with adjustment for sex, ethnicity, place of residence, and residence at birth and stratification by time period. Based on this evaluation it was decided to include the interaction between sex and age and to stratify by place of residence (as this variable was only an adjustment variable). The main effect of sex was estimated in the model without these two changes.

Supplementary Table S7: Number of congenital heart disease (CHD) diagnoses, by ICD-8 and ICD-10 codes[a] by sex, in Greenland and Denmark combined

	Boys/men	Girls/women
**ICD-8 and ICD-10 diagnoses**	N	N
746: Congenital anomalies of heart	967	921
747: Other congenital anomalies of circulatory system	251	238
DI42: Cardiomyopathy	61	26
DQ20: Congenital malformations of cardiac chambers and connections	379	587
DQ21: Congenital malformations of cardiac septa	5 298	6 303
DQ22: Congential malformations of pulmonary and tricuspid valves	570	848
DQ23: Congenital malformations of aortic and mitral valves	1 426	1 202
DQ24: Other congenital malformations of heart	2 824	2 725
DQ25: Congenital malformations of great arteries	1 786	1 988
DQ26: Congenital malformations of great veins	76	75

For specific included ICD-codes, see Supplementary Table S2. [a]Only diagnoses with >50 cases are presented.

Supplementary Figures S1 and S2:

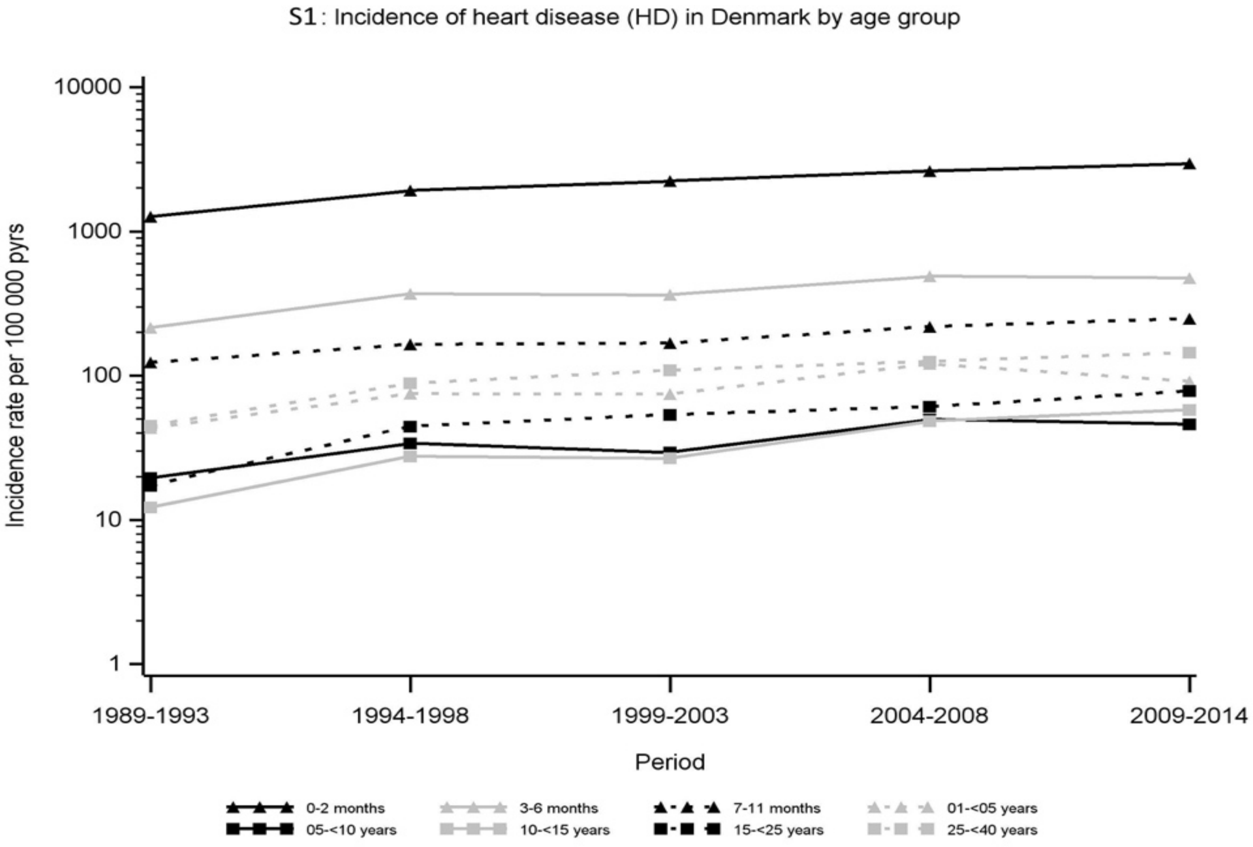

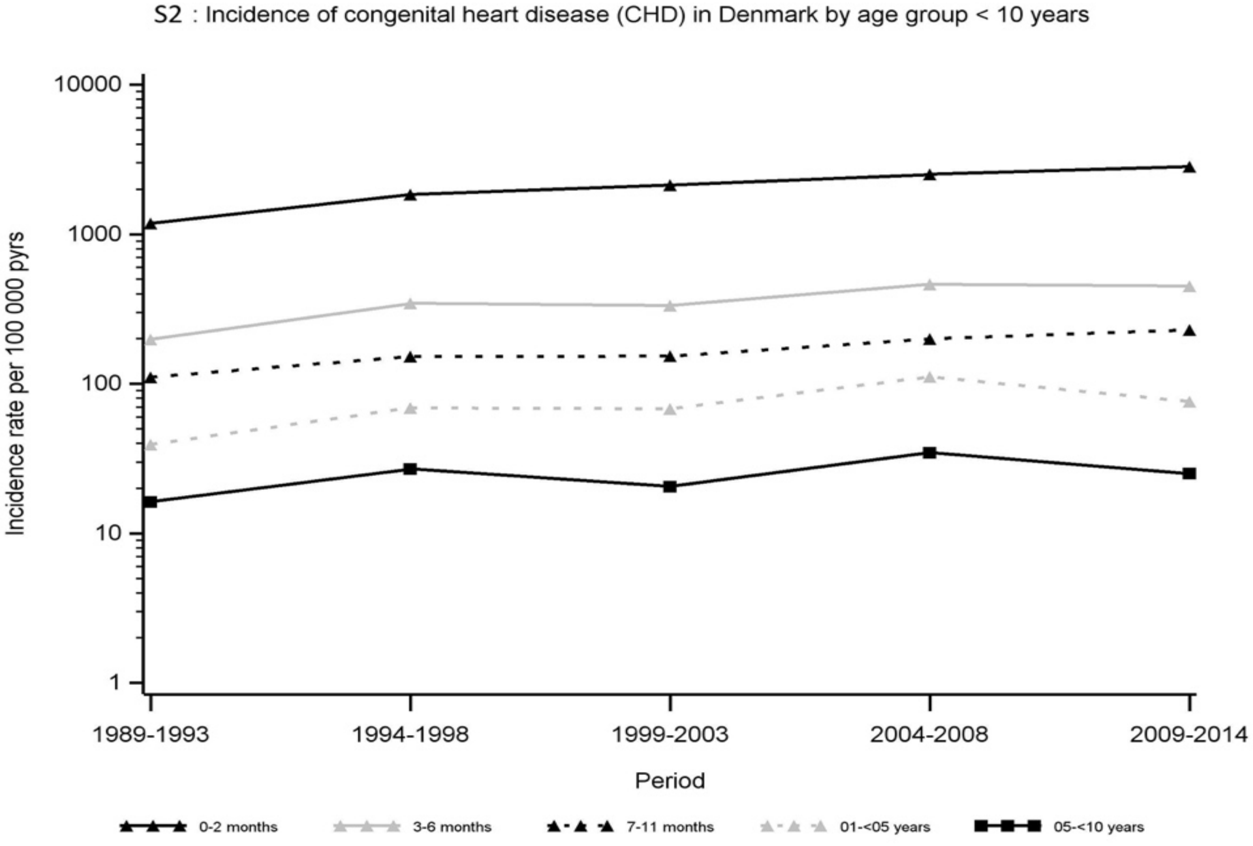

Supplementary Figures S1 and S2: Observed incidence rates (IRs) of hospitalisation in Denmark due to heart disease (S1) and congenital heart disease (S2) during the period 1989-2014. IRs (per 100 000 person years (pyrs)) are illustrated as point estimates in four-year intervals on a logarithmic scale by age groups.

Supplementary Table S8: Number (N) of heart disease (HD) diagnoses[a] by ICD-8 and ICD-10 codes, country, and region

ICD-8 and ICD-10 diagnoses	HD diagnoses given in the capital region of Greenland N	HD diagnoses given outside the capital region of Greenland N	HD diagnoses given in the capital region of Denmark N	HD diagnoses given outside the capital region of Denmark N
394: Diseases of mitral valve	<5[b]	13	38	56
395: Diseases of aortic valve	0	7	44	57
410: Acute myocardial infarction	0	7	154	432
427: Symptomatic heart disease	9	29	536	1 232
746: Congenital anomalies of heart	10	59	567	1 252
747: Other congenital anomalies of circulatory system	<5[b]	12	134	342
DI20: Angina Pectoris	6	14	1 768	3 126
DI21: Acute myocardial infarction	6	12	1 087	2 394
DI25: Chronic ischaemic heart disease	<5[b]	15	464	1 009
DI33: Acute and sub-acute endocarditis	0	8	188	261
DI34: Non-rheumatic mitral valve disorders	<5[b]	23	292	793
DI35: Non-rheumatic aortic valve disorders	5	23	254	998
DI42: Cardiomyopathy	10	16	496	1 306
DI44: Atrioventricular and left bundle-branch block	<5[b]	7	303	603
DI45: Other conduction disorders	16	21	813	1 682
DI46: Cardiac arrest	5	9	491	1 382
DI47: Paroxysmal tachycardia	9	62	3 638	7 241
DI48: Atrial fibrillation and flutter	7	15	1 567	2 533
DI50: Heart failure	<5[b]	9	495	837
DI51: Complications and ill-defines descriptions of heart disease	<5[b]	7	155	225
DQ21: Congenital malformations of cardiac septa	49	132	3 392	8 028
DQ22: Congenital malformations of pulmonary and tricuspid valves	<5[b]	6	493	918
DQ23: Congenital malformations of aortic and mitral valves	<5[b]	16	892	1 718
DQ24: Other congenital malformations of heart	<5[b]	17	1 174	4 357
DQ25: Congenital malformations of great arteries	5	38	1 034	2 697

For specific included ICD-codes, see Supplementary Table S2. [a]Only diagnoses with ≥ 5 cases outside the capital are presented in the table. [b]Due to restrictions, exact N for <5 cases cannot be presented.

Supplementary Table S9: Crude incidence rates (IRs) and adjusted Hazard Ratios (HRs) of hospitalisation for heart disease (HD)[a] when excluding outpatient diagnoses, by demographic characteristics among individuals living in Greenland or Denmark aged 0-<40 years from 1989 through 2014

	Greenland				Denmark			
	Person years	Events	IR[b]	HR[c]	Person years	Events	IR[b]	HR[c]
**Characteristics**	N	N	(95% CI)	(95% CI)	N	N	(95% CI)	(95% CI)
**All**	941 133	647	68.75 (63.65-74.25)		71 245 558	31 848	44.70 (44.21-45.20)	
**Sex overall[c]**								
Girls/women overall	450 007	308	68.44 (61.21-76.53)	1 (ref)	34 918 203	13 901	39.81 (39.15-40.48)	1 (ref)
Boys/men overall	491 126	339	69.03 (62.05-76.78)	1.02 (0.88-1.20)	36 327 355	17 947	49.40 (48.69-50.13)	1.24 (1.22-1.27)
**Sex by age (days/years)[c]**								
**≤6000 days/16.4 years**								
Girls/women				1 (ref)				1 (ref)
Boys/men				0.86 (0.69-1.06)				1.02 (0.99-1.06)
**>6000 days/ 16.4 years**								
Girls/women				1 (ref)				1 (ref)
Boys/men				1.25 (1.00-1.58)				1.41 (1.37-1.45)
**Ethnicity[c]**								
Inuit/mixed[d]	860 365	610	70.90 (65.49-76.76)	1.76 (1.07-2.92)	358 440	205	57.19 (49.88-65.58)	1.26 (1.08-1.48)
Non-Inuit[e]	80 768	37	45.81 (33.19-63.23)	1 (ref)	70 887 118	31 643	44.64 (44.15-45.13)	1 (ref)
**Place of residence at time of Diagnosis[c]**								
Capital (Nuuk /Capital region of Denmark)	239 112	133	55.62 (46.93-65.93)	0.80 (0.66-0.97)	22 007 393	10 135	46.05 (45.16-46.96)	0.98 (0.96-1.00)
Town (excluding Nuuk/Capital region of Denmark)	702 021	514	73.22 (67.15-79.83)	1 (ref)	49 238 165	21 713	44.10 (43.52-44.69)	1 (ref)
**Age at time of diagnosis**								
0-2 months	6 214	117	1 882.7 (1 570.7-2 256.8)		413 311	5 937	1436.4 (1400.4-1473.5)	
3-6 months	6 248	26	416.14 (283.3-611.2)		418 299	823	196.75 (183.76-210.66)	
7-11 months	12 344	37	299.73 (217.2-413.7)		831 079	829	99.75 (93.19-106.78)	
1-4 years	99 502	66	66.33 (52.1-84.4)		6 693 485	2 296	34.30 (32.93-35.73)	
5-9 years	122 937	38	30.91 (22.5-42.5)		8 230 278	1 085	13.18 (12.42-13.99	
10-14 years	118 274	42	35.51 (26.2-48.1)		8 204 277	1 026	12.51 (11.76-13.29)	
15-24 years	211 466	74	34.99 (27.9-44.0)		17 298 249	4 031	23.30 (22.59-24.03)	
25-<40 years	364 148	247	67.83 (59.9-76.9)		29 156 580	15 821	54.26 (53.42-55.11)	

Sensitivity analysis: excluding outpatient diagnoses
[a]Heart disease (HD) is defined as any registered HD ICD code, all available in-patient diagnoses are included. For specific ICD-codes, see Supplementary Table S2. [b]Incidence rate (IR) relates to the crude incidence of HD per 100 000 person years . [c]Hazard ratio (HR) relates to the risk of having HD. [c]Hazard ratios (HRs) relate to the risk of having HD. The assumption was evaluated in a Cox regression model with age as underlying time scale, with adjustment for sex, ethnicity, place of residence, and residence at birth and stratification by time period. Based on this evaluation it was decided to include the interaction between sex and age and to stratify by place of residence (as this variable was only an adjustment variable). The main effect of sex was estimated in the model without these two changes. [d]At least one parent is born in Greenland. [e]None of the parents are born in Greenland.

Supplementary Table S10: Crude incidence rates (IRs) and adjusted Hazard Ratios (HRs) of hospitalisation for congenital heart disease (CHD)[a] when excluding outpatient diagnoses, by demographic characteristics among individuals living in Greenland and Denmark aged 0-<40 years from 1989 through 2014

	Greenland				Denmark			
**Characteristics**	Person years N	Events N	IR[b] (95% CI)	HR[c] (95% CI)	Person years N	Events N	IR[b] (95% CI)	HR[c] (95% CI)
**All**	941 133	302	32.09 (28.67-35.92)		71 245 558	12 740	17.88 (17.57-18.20)	
**Sex overall[c]**								
Girls/women overall	450 007	170	37.78 (32.50-43.91)	1 (ref)	34 918 203	6 604	18.91 (18.46-19.37)	1 (ref)
Boys/men overall	491 126	132	26.88 (22.66-31.88)	0.74 (0.59-0.94)	36 327 355	6 136	16.89 (16.47-17.32)	0.89 (0.86-0.92)
**Sex by age (days/years)[c]**								
**≤1100 days/ 3.0 years**								
Girls/women				1 (ref)				1 (ref)
Boys/men				0.79 (0.59-1.04)				1.03 (0.99-1.07)
**>1100 days/ 3.0 years**								
Girls/women				1 (ref)				1 (ref)
Boys/men				0.69 (0.47-1.02)				0.66 (0.63-0.71)
**Ethnicity[c]**								
Inuit/mixed[d]	860 365	294	34.17 (30.48-38.31)	2.58 (1.19-5.62)	358 440	89	24.83 (20.17-30.56)	1.29 (1.04-1.60)
Non-Inuit[e]	80 768	8	9.90 (4.95-19.81)	1 (ref)	70 887 118	12 651	17.85 (17.54-18.16)	1 (ref)
**Place of residence at time of Diagnosis[c]**								
Capital (Nuuk /Capital region of Denmark)	239 112	57	23.84 (18.39-30.90)	0.81 (0.61-1.09)	22 007 393	4 116	18.70 (18.14-19.28)	1.01 (0.98-1.05)
Town (excluding Nuuk/Capital region of Denmark)	702 021	245	34.90 (30.79-39.55)	1 (ref)	49 238 165	8 624	17.51 (17.15-17.89)	1 (ref)
**Age at time of diagnosis**								
0-2 months	6214	110	1 770.1 (1 468.4-2 133.8)		413 311	5 621	1 360.0 (1 324.9-1 396.0)	
3-6 months	6248	24	384.13 (257.47-573.10)		418 299	747	178.58 (166.22-191.86)	
7-11 months	12 344	33	267.33 (190.05-376.03)		831 079	742	89.28 (83.08-95.94)	
1-4 years	99 502	56	56.28 (43.31-73.13)		6 693 485	2 018	30.15 (28.86-31.49)	
5-9 years	122 937	28	22.78 (15.73-32.99)		8 230 278	815	9.90 (9.25-10.61)	
10-14 years	118 274	12	10.15 (5.76-17.87)		8 204 277	447	5.45 (4.97-5.98)	
15-24 years	211 466	15	7.09 (4.28-11.77)		17 298 249	755	4.36 (4.06-4.69)	
25->40 years	364 148	24	6.59 (4.42-9.83)		29 156 580	1 595	5.47 (5.21-5.75)	

Sensitivity analysis: excluding outpatient diagnoses.
[a]Congenital heart disease (CHD) is defined as the first registered CHD ICD code, irrespectively of other subsequent heart disease diagnosis. All available in-patient diagnoses are included. For specific ICD-codes, see Supplementary Table S2. [b]Incidence rate (IR) relates to the crude incidence of CHD per 100 000 person years [c]Hazard ratio (HR) relates to the risk of having CHD. The assumption was evaluated in a Cox regression model with age as underlying time scale, with adjustment for sex, ethnicity, place of residence, and residence at birth and stratification by time period. Based on this evaluation it was decided to include the interaction between sex and age and to stratify by place of residence (as this variable was only an adjustment variable). The main effect of sex was estimated in the model without these two changes. [d]At least one parent is born in Greenland. [e]None of the parents are born in Greenland.

Supplementary Table S11: Crude incidence rates (IRs) and adjusted Hazard Ratios (HRs) of hospitalisation for heart disease (HD) when including death before age<31 days[a] as heart disease (HD)[b], by demographic characteristics among individuals living in Greenland or Denmark aged 0->40 years from 1989 through 2014

	Greenland				Denmark			
	Person years	Events	IR[c]	HR[d]	Person years	Events	IR[c]	HR[d]
**Characteristics**	N	N	(95% CI)	(95% CI)	N	N	(95% CI)	(95% CI)
**All**	940 742	1 019	108.32 (101.87-115.18)		71 034 197	67 595	95.16 (94.44-95.88)	
**Sex overall[d]**								
Girls/women overall	449 798	481	106.94 (97.79-116.93)	1 (ref)	34 814 379	31 242	89.74 (88.75-90.74)	1 (ref)
Boys/men overall	490 943	538	109.58 (100.71-119.25)	1.05 (0.92-1.18)	36 219 818	36 353	100.37 (99.34-101.40)	1.12 (1.11-1.14)
**Sex by age (days/years)[d]**								
**≤6000 days/ 16.4 years**								
Girls/women				1 (ref)				1 (ref)
Boys/men				0.98 (0.85-1.14)				(1.02-1.07)
**>6000 days/ 16.4 years**								
Girls/women				1 (ref)				1 (ref)
Boys/men				1.21 (0.97-1.51)				1.19 (1.17-1.21)
**Ethnicity[d]**								
Inuit/mixed[e]	860 051	964	112.09 (105.23-119.39)	1.55 (1.08-2.23)	357 136	424	118.72 (107.94-130.58)	1.20 (1.08-1.33)
Non-Inuit[f]	80 690	55	68.16 (52.33-88.78)	1 (ref)	70 677 061	67 171	95.04 (94.32-95.76)	1 (ref)
**Place of residence at time of Diagnosis[d]**								
Capital (Nuuk /Capital region of Denmark)	238 960	165	69.05 (59.28-80.43)	0.61 (0.52-0.72)	21 944 364	21 178	96.51 (95.22-97.82)	0.95 (0.94-0.97)
Town (excluding Nuuk/Capital region of Denmark)	701 781	854	121.69 (113.80-130.13)	1 (ref)	49 089 833	46 417	94.56 (93.70-95.42)	1 (ref)
**Age at time of diagnosis**								
0-2 months	6214	449	7 226.1 (6 587.7-7 926.4)		412 804	14 206	3 441.3 (3 385.2-3 498.4)	
3-6 months	6247	29	464.23 (322.60-668.03)		417 388	1 607	385.01 (366.64-404.31)	
7-11 months	12 342	36	291.69 (210.41-404.38)		828 936	1 547	186.62 (177.55-196.16)	
1-4 years	99 459	75	75.41 (60.14-94.56)		6 671 205	5 463	81.89 (79.75-84.09)	
5-9 years	122 887	42	34.18 (25.26-46.25)		8 199 675	3 019	36.82 (35.53-38.16)	
10-14 years	118 226	47	39.75 (29.87-52.91)		8 178 609	2 986	36.51 (35.22-37.84)	
15-24 years	211 382	80	37.85 (30.40-47.12)		17 256 885	8 859	51.34 (50.28-52.42)	
25-<40 years	363 987	261	71.71 (63.51-80.95)		29 068 697	29 908	102.89 (101.73-104.06)	

Sensitivity analysis: including death before age 31 days as heart disease (HD)
[a]Death before age 31 days is included as HD, unless they have received another diagnosis. [b]HD is defined as any registered HD ICD code, all available in-patient and out-patient diagnoses are included. For specific ICD-codes, see Supplementary Table S2. [c]Incidence rate (IR) relates to the crude incidence of HD per 100 000 person years . [d]Hazard ratio (HR) relates to the risk of having HD. The assumption was evaluated in a Cox regression model with age as underlying time scale, with adjustment for sex, ethnicity, place of residence, and residence at birth and stratification by time period. Based on this evaluation it was decided to include the interaction between sex and age and to stratify by place of residence (as this variable was only an adjustment variable). The main effect of sex was estimated in the model without these two changes. [e]At least one parent is born in Greenland. [f]None of the parents are born in Greenland.

Supplementary Table S12: Crude incidence rates (IRs) and adjusted Hazard Ratios (HRs) of hospitalisation for congenital heart disease (CHD)[b] when including death before age<31 days[a] as CHD, by demographic characteristics among individuals living in Greenland or Denmark aged 0-<40 years from 1989 through 2014

	Greenland				Denmark			
	Person years	Events	IR[c]	HR[d]	Person years	Events	IR[c]	HR[d]
**Characteristics**	N	N	(95% CI)	(95% CI)	N	N	(95% CI)	(95% CI)
**All**	940 742	653	69.41 (64.29-74.95)		71 034 197	29 679	41.78 (41.31-42.26)	
**Sex overall[d]**								
Girls/women overall	449 798	333	74.03 (66.49-82.43)	1 (ref)	34 814 379	15 089	43.34 (42.66-44.04)	1 (ref)
Boys/men overall	490 943	320	65.18 (58.42-72.73)	0.92 (0.79-1.07)	36 219 818	14 590	40.28 (39.63-40.94)	0.92 (0.90-0.95)
**Sex by age (days/years)[d]**								
**≤800 days/ 2.2 years**								
Girls/women				1 (ref)				1 (ref)
Boys/men				0.97 (0.82-1.15)				1.04 (1.01-1.07)
**>800 days/ 2.2 years**								
Girls/women				1 (ref)				1 (ref)
Boys/men				0.74 (0.52-1.06)				0.75 (0.73-0.78)
**Ethnicity[d]**								
Inuit/mixed[e]				1.57 (1.03-2.40)				1.23 (1.07-1.42)
Non-Inuit[f]				1 (ref)				1 (ref)
**Place of residence at time of Diagnosis[d]**								
Capital (Nuuk /Capital region of Denmark)	238 960	85	35.57 (28.76-44.00)	0.51 (0.40-0.64)	21 944 364	8 610	39.24 (38.42-40.07)	0.87 (0.85-0.89)
Town (excluding Nuuk/Capital region of Denmark)	701 781	568	80.94 (74.55-87.87)	1 (ref)	49 089 833	21 069	42.92 (42.34-43.50)	1 (ref)
**Age at time of diagnosis**								
0-2 months	6214	442	7 113.5 (6 480.3-7 808.5)		412 804	13 783	3 338.9 (3 283.6-3 395.1)	
3-6 months	6247	27	432.21 (296.40-630.25)		417 388	1 504	360.34 (342.58-379.02)	
7-11 months	12 342	32	259.28 (183.36-366.65)		828 936	1 413	170.46 (161.80-179.58)	
1-4 years	99 459	64	64.35 (50.37-82.21)		6 671 205	4 896	73.39 (71.36-75.47)	
5-9 years	122 887	31	25.23 (17.74-35.87)		8 199 675	2 057	25.09 (24.03-26.19)	
10-14 years	118 226	14	11.84 (7.01-19.99)		8 178 609	1 226	14.99 (14.17-15.85)	
15-24 years	211 382	18	8.52 (5.37-13.52)		17 256 885	1 612	9.34 (8.90-9.81)	
25-<40 years	363 987	25	6.87 (4.64-10.16)		29 068 697	3 188	10.97 (10.59-11.35)	

Sensitivity analysis: including death before age 31 days as CHD

[a]Death before age 31 days is included as CHD, unless they have received another diagnosis. [b]CHD is defined as the first registered CHD ICD code, irrespectively of other subsequent heart disease diagnosis. All available in-patient and out-patient diagnoses are included. For specific ICD-codes, see Supplementary Table S2. [c]Incidence rate (IR) relates to the crude incidence of CHD per 100 000 person years. [d]Hazard ratio (HR) relates to the risk of having CHD. The assumption was evaluated in a Cox regression model with age as underlying time scale, with adjustment for sex, ethnicity, place of residence, and residence at birth and stratification by time period. Based on this evaluation it was decided to include the interaction between sex and age and to stratify by place of residence (as this variable was only an adjustment variable). The main effect of sex was estimated in the model without these two changes. [e]At least one parent is born in Greenland. [f]None of the parents are born in Greenland.

Supplementary Table S13: Crude incidence rates (IRs) and adjusted Hazard Ratios (HRs) of hospitalisation for heart disease (HD)[a] when excluding outpatient diagnoses and including death before age<31 days[b] as HD, by demographic characteristics among individuals living in Greenland or Denmark aged 0-<40 years from 1989 through 2014

	Greenland				Denmark			
	Person years	Events	IR[c]	HR[d]	Person years	Events	IR[c]	HR[d]
**Characteristics**	N	N	(95% CI)	(95% CI)	N	N	(95% CI)	(95% CI)
**All**	941 132	976	103.70		71 245 548	36 903	51.80	
			(97.40-110.42)				(51.27-52.33)	
**Sex overall[d]**								
Girls/women overall	450 006	460	102.22	1 (ref)	34 918 198	16 117	46.16	1 (ref)
			(93.29-112.00)				(45.45-46.87)	
Boys/men overall	491 126	516	105.06	1.05	36 327 349	20 786	57.22	1.24
			(96.38-114.53)	(0.92-1.19)			(56.45-58.00)	(1.22-1.27)
**Sex by age (days/years)[d]**								
**≤ 1800 days/ 4.9 years**								
Girls/women				1 (ref)				1 (ref)
Boys/men				0.95				1.09
**>1800 days/ 4.9 years**				(0.81-1.12)				(1.05-1.12)
Girls/women				1 (ref)				1 (ref)
Boys/men				1.21				1.36
				(0.99-1.48)				(1.32-1.39)
**Ethnicity[d]**								
Inuit/mixed[e]	860 365	921	107.05	1.41	358 440	234	65.28	1.21
			(100.35-114.19)	(0.98-2.02)			(57.43-74.21)	(1.05-1.40)
Non-Inuit[f]	80 767	55	68.10	1 (ref)	70 887 108	36 669	51.73	1 (ref)
			(52.28-88.70)				(51.20-52.26)	
**Place of residence at time of Diagnosis[d]**								
Capital (Nuuk /Capital region of Denmark)	239 112	152	63.57	0.58	22 007 390	11 609	52.75	0.97
			(54.23-74.52)	(0.49-0.70)			(51.80-53.72)	(0.94-0.99)
Town (excluding Nuuk/Capital Region of Denmark)	702 020	824	117.38	1 (ref)	49 238 158	25 294	51.37	1 (ref)
			(109.63-125.67)				(50.74-52.01)	
**Age at time of diagnosis**								
0-2 months	6 214	446	7 177.7		413 301	10 992	2 659.6	
			(6 541.5-7 875.7)				(2 610.3-2 709.8)	
3-6 months	6 248	26	416.14		418 299	823	196.75	
			(283.34-611.19)				(183.76-210.66)	
7-11 months	12 344	37	299.73		831 079	829	99.75	
			(217.17-413.69)				(93.19-106.78)	
1-4 years	99 502	66	66.33		6 693 485	2 296	34.30	
			(52.11-84.43)				(32.93-35.73)	
5-9 years	122 937	38	30.91		8 230 278	1 085	13.18	
			(22.49-42.48)				(12.42-13.99)	
10-14 years	118 274	42	35.51		8 204 277	1 026	12.51	
			(26.24-48.05)				(11.76-13.29)	
15-24 years	211 466	74	34.99		17 298 249	4 031	23.30	
			(27.86-43.95)				(22.59-24.03)	
25-<40 years	364 148	247	67.83		29 156 580	15 821	54.26	
			(59.88-76.84)				(53.42-55.11)	

Sensitivity analysis: excluding outpatient diagnoses and including death before age 31 days as heart disease (HD).
[a]HD is defined as any registered HD ICD code, all available in-patient diagnoses are included. For specific ICD-codes, see Supplementary Table S2. [b]Death before age 31 days is included as HD, unless they have received another diagnosis. [c]Incidence rate (IR) relates to the crude incidence of HD per 100 000 person years. [d]Hazard ratio (HR) relates to the risk of having HD. The assumption was evaluated in a Cox regression model with age as underlying time scale, with adjustment for sex, ethnicity, place of residence, and residence at birth and stratification by time period. Based on this evaluation it was decided to include the interaction between sex and age and to stratify by place of residence (as this variable was only an adjustment variable). The main effect of sex was estimated in the model without these two changes. [e]At least one parent is born in Greenland. [f]None of the parents are born in Greenland.

Supplementary Table S14: Crude incidence rates (IRs) and adjusted Hazard Ratios (HRs) of hospitalisation for congenital heart disease (CHD)[a] when excluding outpatient diagnoses and including death before age<31 days[b] as CHD, by demographic characteristics among individuals living in Greenland or Denmark aged 0-3<40 years from 1989 through 2014

	Greenland				Denmark			
	Person years	Events	IR[c]	HR[d]	Person years	Events	IR[c]	HR[d]
**Characteristics**	N	N	(95% CI)	(95% CI)	N	N	(95% CI)	(95% CI)
**All**	941 132	631	67.05 (62.01-72.49)		71 245 548	17 801	24.99 (24.62-25.36)	
**Sex overall[d]**								
Girls/women overall	450 006	322	71.55 (64.15-79.81)	1 (ref)	34 918 198	8 822	25.26 (24.74-25.80)	1 (ref)
Boys/men overall	491 126	309	62.92 (56.28-70.34)	0.92 (0.78-1.07)	36 327 349	8 979	24.72 (24.21-25.23)	0.97 (0.94-1.00)
**Sex by age (days/years)[d]**								
**≤150 days/ 0.4 years**								
Girls/women				1 (ref)				1 (ref)
Boys/men				1.03 (0.86-1.24)				1.13 (1.09-1.17)
**>150 days/ 0.4 years**								
Girls/women				1 (ref)				1 (ref)
Boys/men				0.68 (0.50-0.92)				0.75 (0.71-0.79)
**Ethnicity[d]**								
Inuit/mixed[e]	860 365	605	70.32 (64.93-76.15)	1.50 (0.98-2.28)	358 440	118	32.92 (27.49-39.43)	1.20 (1.00-1.45)
Non-Inuit[f]	80 767	26	32.19 (21.92-47.28)	1 (ref)	70 887 108	17 683	24.95 (24.58-25.32)	1 (ref)
**Place of residence at time of Diagnosis[d]**								
Capital (Nuuk /Capital region of Denmark)	239 112	76	31.78 (25.38-39.80)	0.46 (0.36-0.59)	22 007 390	5 591	25.41 (24.75-26.08)	0.97 (0.94-1.01)
Town (excluding Nuuk/Capital Region of Denmark)	702 020	555	79.06 (72.75-85.92)	1 (ref)	49 238 158	12 210	24.80 (24.36-25.24)	1 (ref)
**Age at time of diagnosis**								
0-2 months	6 214	439	7 065.0 (6 434.1-7 757.8)		413 301	10 682	2 584.6 (2 536.0-2 634.0)	
3-6 months	6 248	24	384.13 (257.47-573.10)		418 299	747	178.58 (166.22-191.86)	
7-11 months	12 344	33	267.33 (190.05-376.03)		831 079	742	89.28 (83.08-95.94)	
1-4 years	99 502	56	56.28 (43.31-73.13)		6 693 485	2 018	30.15 (28.86-31.49)	
5-9 years	122 937	28	22.78 (15.73-32.99)		8 230 278	815	9.90 (9.25-10.61)	
10-14 years	118 274	12	10.15 (5.76-17.87)		8 204 277	447	5.45 (4.97-5.98)	
15-24 years	211 466	15	7.09 (4.28-11.77)		17 298 249	755	4.36 (4.06-4.69)	
25-<40 years	364 148	24	6.59 (4.42-9.83)		29 156 580	1 595	5.47 (5.21-5.75)	

Sensitivity analysis: excluding outpatient diagnoses and including death before age 31 days as CHD.
[a]CHD is defined as first registered CHD ICD code, all available in-patient diagnoses are included. For specific ICD-codes, see Supplementary Table S2. [b]Death before age 31 days is included as CHD, unless they have received another diagnosis. [c]Incidence rate (IR) relates to the crude incidence of CHD per 100 000 person years. [d]Hazard ratio (HR) relates to the risk of having CHD. The assumption was evaluated in a Cox regression model with age as underlying time scale, with adjustment for sex, ethnicity, place of residence, and residence at birth and stratification by time period. Based on this evaluation it was decided to include the interaction between sex and age and to stratify by place of residence (as this variable was only an adjustment variable). The main effect of sex was estimated in the model without these two changes. [e]At least one parent is born in Greenland. [f]None of the parents are born in Greenland.
